# IQGAP3 Is an Important Mediator of Skin Inflammatory Diseases

**DOI:** 10.3390/ijms25084545

**Published:** 2024-04-21

**Authors:** Alena Zolotarenko, Sergey Bruskin

**Affiliations:** Laboratory of Functional genomics, Vavilov Institute of General Genetics Russian Academy of Sciences, 119991 Moscow, Russia

**Keywords:** keratinocytes, IQGAP3, RNAseq, inflammation, psoriasis, pathway analysis, proliferation

## Abstract

IQGAP3 (IQ Motif Containing GTPase Activating Protein 3) is member of the IQGAP family of scaffold proteins, which are essential for assembling multiprotein complexes that coordinate various intracellular signaling pathways. Previous research has shown that *IQGAP3* is overexpressed in psoriatic skin lesions. Given its involvement in processes like cell proliferation and chemokine signaling, we sought to explore its molecular role in driving the psoriatic phenotype of keratinocytes. By conducting transcriptome profiling of HaCaT keratinocytes, we identified numerous psoriasis-associated pathways that were affected when *IQGAP3* was knocked down. These included alterations in NFkB signaling, EGFR signaling, activation of p38/MAPK and ERK1/ERK2, lipid metabolism, cytokine production, and the response to inflammatory cytokine stimulation. Real-time analysis further revealed changes in cell growth dynamics, including proliferation and wound healing. The balance between cell proliferation and apoptosis was altered, as were skin barrier functions and the production of IL-6 and IFNγ. Despite these significant findings, the diversity of the alterations observed in the knockdown cells led us to conclude that IQGAP3 may not be the best target for the therapeutic inhibition to normalize the phenotype of keratinocytes in psoriasis.

## 1. Introduction

Psoriasis is a common inflammatory skin condition involving complex interactions between immune and skin cells. In psoriatic skin, abnormal proliferation of keratinocytes and production of inflammatory cytokines attract activated lymphocytes, leading to the development of characteristic hyperkeratotic lesions called psoriatic plaques. Keratinocytes in plaques produce antimicrobial peptides, cytokines, and chemokines, which can promote their own proliferation and attract additional immune cells. These cytokines, including IL-17, IL-12, IL-22, IL-23, TNFα, and IFNγ, play a key role in the development and maintenance of psoriatic plaques [1]. Over 80 psoriasis susceptibility loci have been identified, indicating a complex genetic basis for the disease [2]. Understanding the inflammatory pathways involved in psoriasis has led to the development of targeted therapies, such as monoclonal antibodies and small molecules, which can inhibit specific immune cell activation or inflammatory pathways. However, the varied response to treatment among patients highlights the need for further investigation into these pathways for the development of personalized treatment approaches [3].

In general, the main target classes of marketed drugs include G protein-coupled receptors (GPCRs), ion channels, kinases and nuclear receptors [4]. Binding of a ligand to a receptor initiates a cascade of reactions mediated by kinases and scaffold proteins, leading to the activation of transcription factors and the expression of the effector target genes. Many of the inflammatory pathways active in psoriasis and triggered by different ligands are mediated by the same kinases or scaffold proteins. For instance, more than 50 cytokines signal via the JAK/STAT pathway to orchestrate inflammation and control the immune response [5] Targeting these key molecules connecting different molecular pathways may offer potential therapeutic strategies to normalize the abnormal characteristics of psoriatic skin cells. Scaffold proteins, although less studied than kinases, seem to be promising candidates for solving this issue. Scaffold proteins play crucial roles in coordinating cellular signaling by facilitating the interactions between signaling proteins or regulating their localization within a cell. In some cases, scaffold proteins can be directly regulated themselves, influencing the activation or inhibition of signaling pathways. Altering the activity of scaffold proteins through the changes in their conformation or localization as well as using small interfering peptides have been shown to be a promising therapeutic strategy in a number of studies [6].

The IQGAP family of scaffold proteins mediate various signaling pathways, including EGFR and WNT pathways, CXCR2 and CXCR4 signaling, IFN and NFkB signaling, the cascades of cytoskeletal organization, cell adhesion and migration [7,8] Most of the aforementioned pathways play roles in the development of psoriatic plaques. Additionally, a hallmark of psoriasis is hyperkeratosis arising due to the hyperproliferation of keratinocytes, and different researchers have shown partial knockdown of IQGAP proteins to decrease the growth and proliferation rates and migration capacity of various types of cells—skin cells [9], liver cells [10], mammary gland cells [11], neurons [12] and other types. Previously, we have found *IQGAP3* to be the only member of this family overexpressed in lesional psoriatic skin [13], and Monteleon and colleagues have found IQGAP3 to be a promising target for skin cancer. This suggests that IQGAP3 knockdown in the skin can be a promising strategy for correcting the excessive proliferation of keratinocytes in psoriasis.

This research aims to investigate the role of IQGAP3 in the development of the psoriatic phenotype in keratinocytes and to explore its potential as a therapeutic target for psoriasis treatment.

## 2. Results

### 2.1. IQGAP3 Knockdown in Keratinocytes

**Figure 1:**
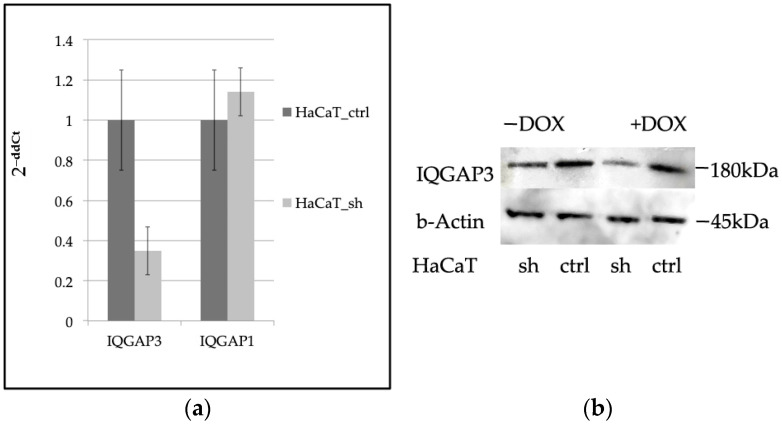
(**a**) qPCR evaluation of the expression levels of *IQGAP3* and *IQGAP1* in HaCaT_sh vs. HaCaT_ctrl, (*n* = 3, mean ± S.D.); (**b**) Western blot evaluation of the expression levels of IQGAP3 in HaCaT_sh vs. HaCaT_ctrl. Anti-IQGAP3 antibodies, H00128239-M01 NovusBio; anti-β-Actin antibodies #4970 Cell Signaling.

To knock down *IQGAP3* in keratinocytes, we developed a keratinocyte cell line called HaCaT_sh by lentivirally transducing with an shRNA-coding vector, pEZ_Tet_pLKO_puro. Initially, we tested several shRNAs from the literature via transient transfection to ensure they did not exhibit off-target activity. Through transient transfection experiments (Figure S1 in the Supplementary File S1), we identified the most effective shRNA for knockdown. Subsequently, we designed corresponding shRNA oligos, cloned into the pEZ_Tet_pLKO_puro_sh vector, and generated lentiviral particles. These particles were then used to transduce HaCaT keratinocytes, and stable clones were selected using puromycin. The expression of the target gene was evaluated in these stable clones. In comparison to control cells (HaCaT_ctrl), which were transduced with a scrambled shRNA vector, *IQGAP3*-depleted cells showed only approximately 35% of the *IQGAP3* mRNA level (Figure 1a). Western blot analysis confirmed the knockdown (Figure 1b). Additionally, considering the high homology between *IQGAP1* and *IQGAP3*, we also examined the expression of *IQGAP1*. qPCR analysis indicated no significant change in *IQGAP1* expression in the knockdown cells (Figure 1a).

### 2.2. The Knockdown of IQGAP3 Significantly Alters the Growth Dynamics of Cells

To assess the impact of the knockdown on the proliferative characteristics and migratory capacity of HaCaT keratinocytes, we employed a real-time evaluation system called xCelligence by Agilent. This technology relies on measuring the electrical impedance resulting from the interaction of a cell’s plasma membrane with a golden electrode at the bottom of the well. Consequently, it enables the dynamic assessment of cell behavior in response to various stimuli or physical influences.

**Figure 2:**
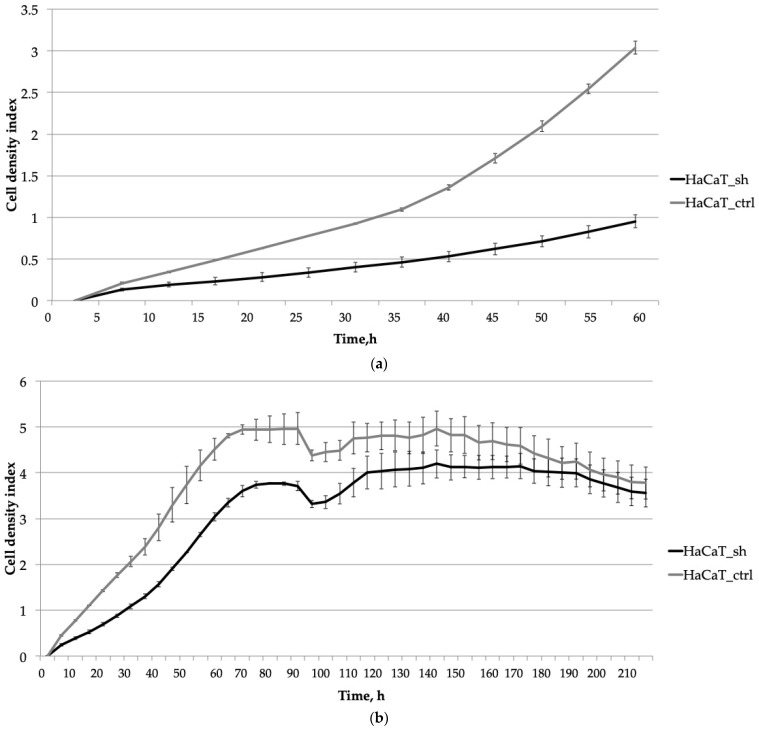
The impact of the *IQGAP3* knockdown on the growth dynamics of HaCaT keratinocytes was assessed at different stages of cultivation: (**a**) Initial stages of cultivation (0–60 h): The curves represent the average cell density of three replicates with standard deviation (±sd). (**b**) Prolonged cultivation until plateau (0–70 h) plus wound healing and cell culture aging (95–210 h): similarly, the curves depict the average cell density of three replicates with standard deviation (±sd).

Initially, we focused on evaluating the early stages of cell cultivation, including adhesion, spreading, and proliferation. Our analysis over a 60-h period revealed that adhesion and spreading (observed within the first 5 h, as depicted in Figure 2a) did not exhibit significant differences between the two cell lines. However, HaCaT_ctrl cells displayed notably faster proliferation and expansion across the culture surface compared to HaCaT_sh cells during the initial stages of culture (Figure 2a).

Extended cell cultivation (Figure 2b) revealed that inhibiting *IQGAP3* led to slower rates of cell proliferation. This was evidenced by a delayed and lower plateau on the growth curve, with the plateau occurring at 75 h compared to 70 h for the control cells.

To evaluate the impact of *IQGAP3* knockdown on wound healing, we conducted a monolayer wound assay at the 95-h time point. The growth curve showed no significant differences in the speed of wound healing or in the dynamics of cell culture aging (Figure 2b, time point 100 h and later).

### 2.3. Knocking down IQGAP3 Reduces the Effect of Pro-Inflammatory Cytokine Stimulation

**Figure 3:**
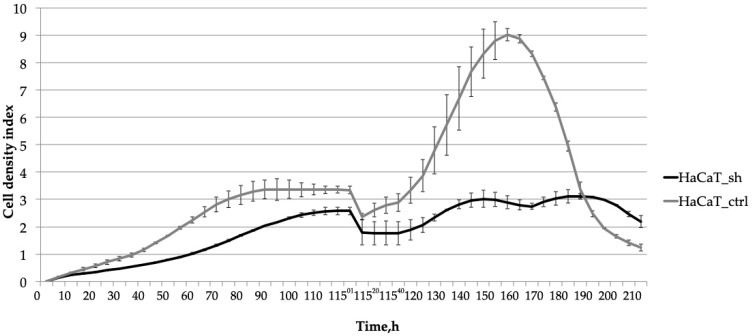
The effect of the *IQGAP3* knockdown on the growth dynamics of HaCaT keratinocytes under inflammatory environment: the cells were cultivated until plateau (0–75 h); the culture was wounded at the time point 115 h and stimulated with inflammatory cytokines (IL-17, TNFa, IFNg) and cultivated for the next 95 h. The curves represent the average cell density of three replicates ± sd.

Subsequent analysis (Figure 3) revealed the impact of the *IQGAP3* knockdown on cellular response to cytokine treatment. The suppression of *IQGAP3* under the pro-inflammatory milieu notably altered the growth dynamics of cells. In control cells, wound healing was accelerated, and the addition of cytokines (IL-17 at 100 ng/mL, TNFα at 25 ng/mL, and IFNγ at 20 ng/mL) stimulated their proliferation, resulting in a denser monolayer. This stimulation was depicted as a bell-shaped curve on the cell density graph, with the peak density occurring at 155 h (60 h post-scratching). Conversely, in HaCaT_sh cells, cytokine treatment failed to elicit a similar stimulatory effect. This suggests that *IQGAP3* knockdown nearly completely inhibited the stimulatory effect induced by the inflammatory cytokines TNFα, IFNγ, and IL-17.

In summary, the inhibition of *IQGAP3* resulted in a notable reduction in the rate of cell proliferation. Furthermore, it nearly completely suppressed the growth-stimulating effects induced by the pro-inflammatory cytokines.

### 2.4. RNA Sequencing Analysis Unveils the Pathways Affected by IQGAP3 Knockdown

**Figure 4:**
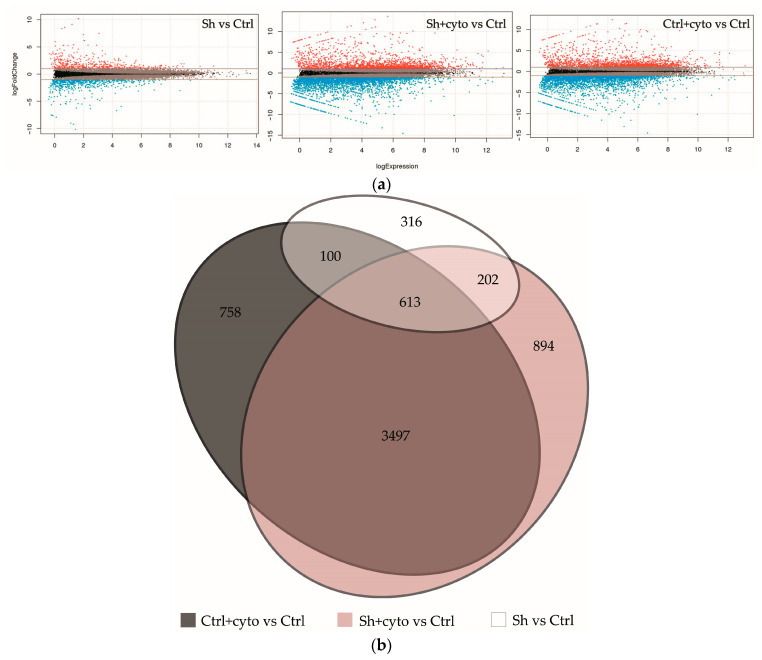
Transcriptomic changes in keratinocytes with *IQGAP3* knockdown. (**a**)—MA plots visualizing log2 fold-change for all of the transcripts detected in each comparison. (**b**)—Venn diagram showing the extent of overlap among the differentially expressed genes with ≥2-fold increase/decrease from each comparison (FDR < 0.01).

We conducted RNA sequencing (RNAseq) of both HaCaT_sh and HaCaT_ctrl cell lines, with and without cytokine stimulation, to elucidate the molecular mechanisms underlying the observed growth alterations and response to cytokines (see Figure 4, the complete list of differentially expressed genes (DEGs) can be found in the Supplementary File S2). The knockdown of *IQGAP3* resulted in significant differential expression (fold change ≥ 2, FDR < 0.01) of 1231 genes, with 529 upregulated and 702 downregulated genes (Sh vs. Ctrl).

Gene ontology enrichment analysis (FDR < 0.05, the full list of gene ontology terms can be found in the Supplementary File S2) revealed that *IQGAP3* knockdown affected various biological pathways, including skin development, epithelial cell differentiation, cell adhesion, migration, and kinase signaling. Downregulated genes were enriched in pathways related to proliferation, keratinocyte differentiation, epidermis development, cell adhesion, inflammation, immune response cascades, and MAPK, JNK, and ERK-kinase signaling. These genes were associated with molecular functions such as calcium ion binding, heparin and cadherin binding, MHC class II signaling, signal transduction from pattern recognition and RAGE receptors, and regulation of calcium channels. On the other hand, genes overexpressed under *IQGAP3* knockdown were involved in IkB/NF-kB signaling, pro-inflammatory activation, biosynthesis of IL-6 and NO, activation of calcium transport, cell adhesion, extracellular matrix organization, and lipid metabolism. Their proteins were found to bind metal ions, particularly calcium, lipopolysaccharides, proteases, and actin, and to regulate the activity of serine-threonine peptidases and to bind ligands to the IL1 receptor, participating in transcriptional regulation.

### 2.5. IQGAP3-Mediated Pathways under Inflammatory Stimulation

Under cytokine stimulation, HaCaT_ctrl exhibited differential expression of 4968 genes (2038 upregulated and 2930 downregulated), while HaCaT_sh showed differential expression of 5206 genes (2108 upregulated and 3098 downregulated). Notably, 4110 of the differentially expressed genes (DEGs) were common to both cell lines (see Figure 4b), and the full list of gene ontology (GO) enrichment for all groups can be found in the Supplementary File S2.

**Figure 5:**
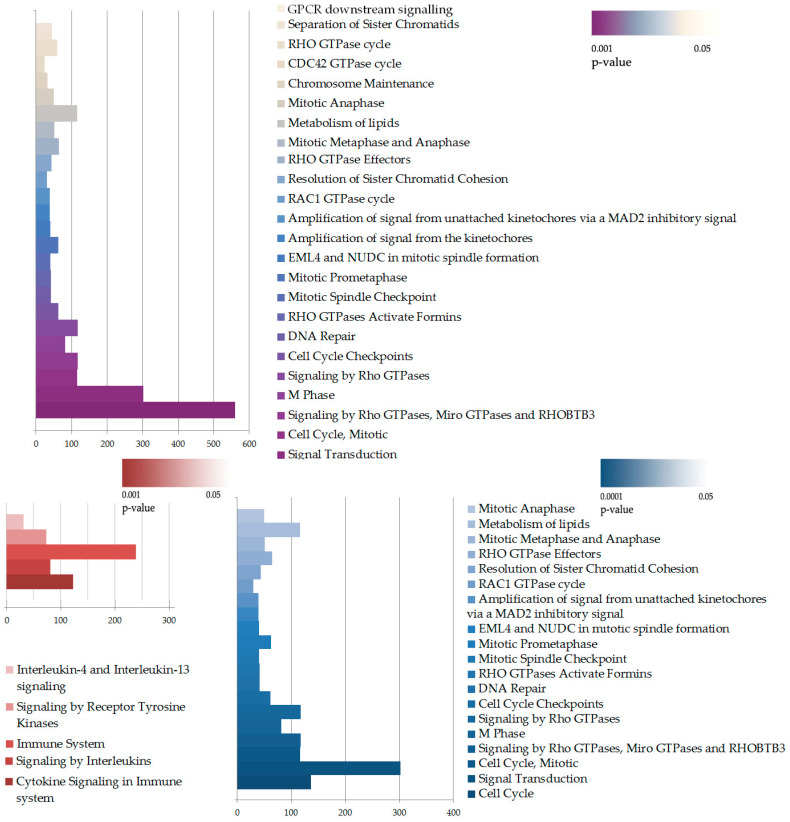
Pathway enrichment analysis of DEGs in HaCaT_sh+cyto vs. HaCaT_ctrl+cyto. The enrichment was performed with complete DEG lists (upper panel) or separately upregulated (red, lower panel) and downregulated (blue, lower panel) DEGs. The size of the bar illustrates number of DEGs in the pathway, while the color indicates *p*-value of the enrichment.

**Figure 6:**
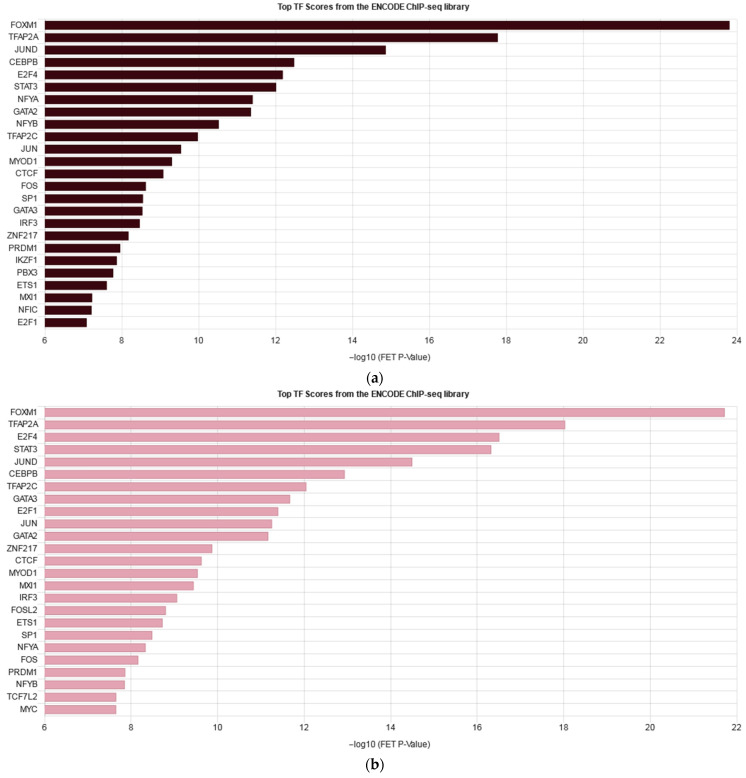
Enrichment bars listing top 25 transcription factors regulating DEGs in HaCaT_ctrl+cyto (**a**) and HaCaT_sh+cyto (**b**) according to the ENCODE project.

Overall, many cascades of inflammation, cell proliferation, and migration were similarly enriched in both cell lines. However, on average, the level of gene expression alterations under cytokine stimulation appeared to be less prominent in the knockdown cells, while the number of differentially expressed genes was slightly higher in the knockdown cells. To further understand the impact of *IQGAP3* knockdown on pathway enrichment, we performed pathway analysis using the Reactome pathway database and compared the lists of the enriched pathways (Figure 5) and ontologies (Figures S2 and S3) as well as the possible transcription factor regulators of the DEGs (Figure 6).

To identify the cytokine-induced pathways affected by *IQGAP3* knockdown and relevant to psoriasis, we conducted a manual analysis. We identified the gene ontologies (GO) enriched in the control cells but not in the knockdown cells, and vice versa. Table 1 presents a selection of GO terms considered important for psoriasis.

**Figure 7:**
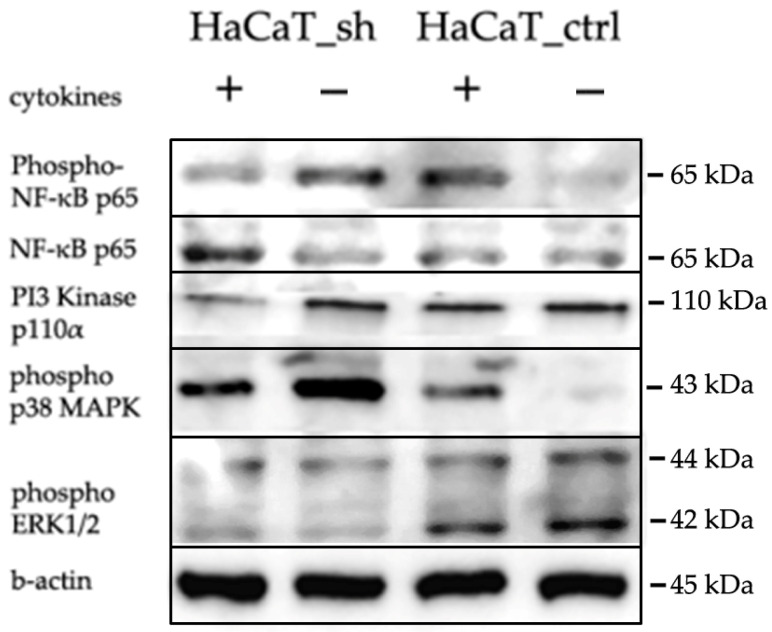
WB analysis of the *IQGAP3* knockdown effects in HaCaT cells following cytokine stimulation (IL-17, TNFa, IFNg). Antibodies: anti-IQGAP3, H00128239-M01 NovusBio; Cell Signaling anti-β-Actin, #4970; anti-NFkB p65, #6956; anti-phospho-NFkB p65, #3033; anti-phospho-p44/42 MAPK (Erk1/2), #4370; anti-phospho-p38 MAPK, #4511; anti-PI3K p110α.

In order to verify our findings, we performed Western blot analysis on several targets important for psoriatic inflammation. Figure 7 shows that *IQGAP3* knockdown itself led to the activation of p38 MAPK and NFkB signaling and inhibition of ERK1/2. However, cytokine stimulation on the knockdown background led to less accumulation of phospho-p65 and pI3K.

## 3. Discussion

IQGAP proteins play crucial roles in regulating cell morphology, motility and signal transduction by interacting with signaling molecules, cell adhesion proteins, and components of the cytoskeleton [12,14,15]. In most cases, IQGAP3 is associated with increased cell proliferation and was found to be overexpressed in various types of cancers [16,17,18,19]. In cancer cells, IQGAP3 promotes cell migration, epithelial to mesenchymal transition (EMT), invasion and metastasis by activating TGF-β signaling [15]. Silencing *IQGAP3* in breast cancer cells inhibits proliferation and metastasis. The effect is associated with p53, MMP9, Snail, CDC42, p-ERK1/2, KIF2C, KIF4A, PCNA and Twist [20]. Co-immunoprecipitation analysis revealed that IQGAP3 promotes cell proliferation through its interaction with PKCδ [21]. Moreover, deletion of *IQGAP3* was found to inhibit cell growth in a 3D Matrigel [22]. *Iqgap3* was recognized as a marker for homeostatic stem cells situated in the isthmus of the stomach epithelium. Utilizing lineage tracing in a mouse model, it was shown that Iqgap3-expressing cells possess stem cell activity [23].

Psoriasis is a skin pathology with excessive proliferation and activation of keratinocytes that partly resembles EMT [24]. Keratinocytes play crucial roles not only in structural and barrier functions of skin, but also as immunocompetent cells involved in the immune response. In psoriatic plaques, keratinocytes exhibit elevated production of pro-inflammatory cytokines and chemotactic factors, along with increased proliferation and decreased differentiation rates [25]. Previous research has shown that *IQGAP3* is overexpressed in the skin of patients with psoriasis [13].

To assess whether IQGAP3 plays a role in the hyperproliferative phenotype of psoriatic keratinocytes, we developed a keratinocyte cell line called HaCaT_sh, which expresses small hairpin RNA targeting *IQGAP3*. Analysis of the growth rate of HaCaT_sh cells revealed slower growth compared to control cells, resulting in a later and lower plateau on the growth curve (Figure 2b, time point 75 h). The wound healing dynamics of the cells remained unchanged, as did the final cell density and the overall dynamics of cell culture aging (Figure 2b, time points 95–220 h). However, the response to pro-inflammatory cytokine stimulation was altered: while control cells exhibited active proliferation and elevated wound healing rates, the knockdown cells showed diminished proliferation upon stimulation (Figure 3, time points 115–170 h). Following cytokine-stimulated overgrowth of the control cells, the final cell density of the culture (Figure 3, time points 175–210 h) was lower compared to the knockdown cells. This could be attributed to nutrient shortages in the cell medium due to the higher cell number, as well as the activation of cell death-associated pathways in the control cells following cytokine stimulation.

To gain deeper insights into the molecular mechanisms underlying the observed growth alterations, we conducted RNA-seq analysis of the knockout and control cells under pro-inflammatory stimulation. *IQGAP3* knockdown itself led to the expression alterations of 1231 genes, which, in line with the growth curve data, enriched the ontologies mainly associated with cell migration and proliferation, innate immunity and inflammatory responses, calcium signaling and lipid metabolism. Western blot analysis had shown that the knockdown led to the background activation of MAPK and NFkB signaling but less phosphorylation of ERK1/2 (Figure 5).

When we analyzed the differences in the cell response to cytokine stimulation (IL17, IFNγ, TNFα—a cocktail mimicking the cytokine background in psoriasis), we observed that the average level of gene expression alterations appeared to be less prominent in the knockdown cells (Figure 4b, Supplementary Files S1 and S2). The analysis revealed that most of the pathways were similarly enriched with differentially expressed genes in both cell lines, and the lists of transcription factors regulating DEGs were not much different (Figure 6, Figures S2 and S3). Western blot analysis had shown that cytokine stimulation on the knockdown background led to less accumulation of phospho-p65 and pI3K (Figure 7). It seems that *IQGAP3* knockdown itself leads to the activation of proinflammatory signaling, but cytokine stimulation attenuates this effect, possibly due to the activation of compensatory mechanisms.

Apparently, in our transcriptome analysis, we did not find any pathways to be completely knocked down. This could be because of the level of *IQGAP3* knockdown as well as due to the nature of IQGAP3 itself: it is neither a receptor nor a transcription factor but a mediator and a coordinator of many signaling nodes in cells, so none of the pathways were abolished, but many were affected. The most significant differences were observed in the enrichment of certain pathway groups, including small GTPase-mediated pathways and cell cycle pathways (Figure 5).

Small GTPases play an important role in the development of the multilayered epidermis and various skin appendages during embryogenesis. They are indispensable for cell spreading and migration, as well as the establishment of epithelial cell–cell contacts, and enhance IQGAP oligomerization [26,27]. Inhibition of GTPase activation leads to significant delays in wound closure [28]. Moreover, the altered growth dynamics and changes in cornification and barrier functions observed in *IQGAP3*-knockdown cells may be directly influenced by dysregulated GTPase signaling pathways.

The differences in the enrichment of cell cycle-associated pathways following *IQGAP3* knockdown may reflect alterations in cellular growth dynamics under inflammatory conditions. Additionally, *IQGAP3* has been identified as a crucial factor in stem cell regulation, being expressed in rapidly proliferating stem cells and cancer cells alike [29,30].

In addition to the aforementioned pathways, *IQGAP3* knockdown was observed to impede several other pathways and ontologies pivotal for the development and progression of psoriasis plaques (Table 1). *IQGAP3* knockdown inhibited EGFR signaling, which plays a crucial role in epidermal homeostasis and regulation of skin inflammatory responses and has elevated expression in active epidermal lesions of psoriasis [31]. Like IQGAP1, IQGAP3 augmented EGF-stimulated activation of EGFR and signaling to ERK, which has been suggested to enhance IQGAP3-driven tumorigenesis [9,32].

Phosphorylation of p38 MAPK is elevated in the affected areas of psoriatic skin, leading to the production of the proinflammatory cytokines like TNFα and antimicrobial peptides such as cathelicidin, β-defensins 2 and 3, S100A7 and S100A8 [33]. In our study, *IQGAP3* inhibition led to the accumulation of phospho-p38 that may contribute to the elevated levels of inflammatory cytokine production.

Similarly to p38, ERK1/2 is activated in psoriatic epidermis, and inhibition of ERK via a specific inhibitor, JSI287, or a negative regulator of the MAPK pathway, DUSP1, significantly inhibited keratinocyte proliferation and promoted apoptosis and reduced IMQ-induced psoriasiform lesion formation [34]. TNFα, IL-17, and IL-22 stimulate ERK phosphorylation, leading to the increased expression of crucial psoriasis mediators such as IL-36α and IL-36γ [35]. In *IQGAP3* knockdown cells, the levels of phospho-ERK decrease significantly [9,36], and our research has shown less phospho-ERK1/2 accumulation both without or with cytokine stimulation.

In summary, the knockdown of *IQGAP3* exhibited a significant anti-psoriatic effect, manifesting in the activation of the pathways of cell adhesion and tight junction development, stimulated cornification, improved skin barrier functions, activated lipid metabolism, and facilitated apoptosis of epithelial cells, crucial for cornified envelope development. Moreover, knockdown influenced immune response pathways, negatively impacted chemotaxis, and suppressed activation of the ERK1 and ERK2 signaling cascades.

Despite the observed decrease in proliferation rates via growth curve analysis, at the molecular level, *IQGAP3* knockdown did not fully abolish the stimulating effect of inflammatory cytokines in cultured keratinocytes. Notably, several pathways, including the positive regulation of interleukin-6 production, epithelial cell migration, MAPK activation and NFkB transcription factor activity, were more prominently activated in the knockout cells compared to the control cells. Given the multitude and diversity of pathways regulated by IQGAP3 in keratinocytes, targeting this protein for therapeutic inhibition may not be the most effective strategy to normalize the phenotype of keratinocytes is psoriasis. More research is needed to evaluate the possibility to target this protein as a part of combination therapy, for example, together with the TNFα blockers or IL-17 inhibitors. Their use may lead to chronic NFkB inhibition, which could result in immunodeficiencies and, in this case, NFkB activation due to *IQGAP3* inhibition may be favorable for the overall homeostasis of skin. Moreover, as research has shown that IQGAP3 inhibition led to genome instability and aneuploidy [30], in the case of therapeutic inhibition, the absence of such negative effects should be carefully verified.

## 4. Materials and Methods

### 4.1. Cell Culture

Immortalized human keratinocyte cell line HaCaT was generously provided by Dr. Vorotelyak, Russia and human embryonic kidney cells HEK293T were generously provided by Dr. M. Lagarkova, Russia. Cells were cultured in Dulbecco’s Modified Eagle Medium (DMEM) (ThermoFisher Scientific, Waltham, MA, USA) supplemented with 10% fetal bovine serum (FBS) (Thermo Fisher Scientific, Waltham, MA, USA), 4 mM of L-Glutamine (Paneco, Moscow, Russia), and 10 U/mL of anti-anti (Gibco, ThermoFisher Scientific, Waltham, MA, USA) at 37 °C and 5% CO_2_, and routinely tested for mycoplasma contamination using MycoReport (Evrogen, Moscow, Russia). The inducible vectors were stimulated by supplementation of the medium with 1000 ng/mL doxycycline (Sigma-Aldrich, Merck KGaA, Darmstadt, Germany) or with a mix of pro-inflammatory cytokines IL17A, TNFα, and IFNγ (Peprotech, ThermoFisher Scientific, Waltham, MA, USA) (IL-17 100 ng/mL; TNFα 25 ng/mL, IFNγ 20 ng/mL).

### 4.2. Oligos

All of the sequences of primers and constructs are listed in the Supplementary Materials Tables S1 and S2.

### 4.3. Lentiviral Transduction

EZ-Tet-pLKO-Puro vector (#85966 Addgene, Watertown, MA, USA) was a gift from Dr. Cindy Miranti’s lab [37]. The shRNA-coding duplex was cloned into EZ-Tet-pLKO-Puro using NheI and EcoRI. The 3rd-generation lentiviral vector system consisting of pMDLG/RRE, pCMV_VSV_G, pRSV/Rev vectors was a gift from Didier Trono (#12251, #8454, #2253, Addgene, Watertown, MA, USA) [38]. Lentiviral particle production was performed in HEK293T. The supernatant containing lentiviral particles was filtered, concentrated using LentiX (Clontech, Takara Bio Inc., Shiga, Japan) and used for MOI evaluation. HaCaT cells in FBS-free medium were transduced in MOI 2:1 and selected on puromycin (Sigma-Aldrich, Merck KGaA, Darmstadt, Germany) for stable clones.

### 4.4. RNA Isolation, Reverse Transcription and Quantitative PCR

In order to quantify IQGAP3 knockdown, HaCaT_sh and HaCaT_ctrl cells were seeded in 3 biological replicates in a complete medium and stimulated with doxycycline (Sigma-Aldrich, Merck KGaA, Darmstadt, Germany) for 72 h. Next, the cells were lysed, and total RNA was isolated using RNEasy mini kit (Qiagen, Hilden, Germany) according to the manufacturer’s instructions. RNA concentration was quantified on a Qubit fluorimeter with the RNA BR Assay Kit (Life Technologies, ThermoFisher Scientific, Waltham, MA, USA), and 1 μg of RNA was transcribed into complementary DNA (cDNA) using SuperScript III Reverse Transcriptase (Invitrogen, ThermoFisher Scientific, Waltham, MA, USA) following the manufacturer’s instructions.

qPCR amplification was performed in a thermal cycler (Eco Real-Time PCR System, Illumina, San Diego, CA, USA) using the two-step program (primary denaturation at 95 °C for 4 min, then 45 cycles: denaturation at 94 °C for 15 s, annealing + elongation at 60 °C for 1 min) in triple technical replicates. The PCR was performed using 5XqPCRmix-HS + ROX (Evrogen, Moscow, Russia). The level of expression of target genes was normalized to 3 housekeeping genes—GAPDH, GusB, and B2M—using the 2^−ΔΔCt^ method. The significance of the differences was evaluated using R software version 3.6.0 and Mann–Whitney U test.

### 4.5. Western Blotting and Antibodies

Wild type HaCaT cells, HaCaT_ctrl, and HaCaT_sh cells were cultured in full DMEM medium supplemented with 10% FBS. Cells were stimulated with 1000 ng/mL doxycycline for 72 h. Subsequently, the cells were trypsinized and collected with RIPA buffer supplemented with Protease Inhibitor Cocktail 4 MammCell/Tissue Plus (#A7757, AppliChem, Darmstadt, Germany). For the WB analysis of HaCaT_sh and HaCaT_ctrl under cytokine stimulation, the cells were initially seeded in complete medium and 4 h later stimulated with doxycycline (Sigma-Aldrich, Merck KGaA, Darmstadt, Germany) for 24 h. Subsequently, the medium was replaced with fresh medium containing both doxycycline and pro-inflammatory psoriatic cytokines (IFNγ, TNFα, IL17) at concentrations of 20 ng/mL, 25 ng/mL, and 100 ng/mL, respectively. After 72 h of stimulation, the cells were trypsinized and collected with RIPA buffer supplemented with Protease Inhibitor Cocktail 4 MammCell/Tissue Plus (#A7757, AppliChem, Darmstadt, Germany). Western blotting was performed following the manufacturer’s instructions with several modifications: the samples were loaded on a 4–12 Bis-Tris gel (40% bis-acrylamide, 1.5M Tris-HCL pH8.8/0.5M tris-HCL pH 6.8, 10% *w*/*v* ammonium persulfate, 0.04% *v*/*v* TEMED, 10% *v*/*v* SDS, and distilled water to reach the final volume) in equal amounts for each of the experiments, typically between 5 and 10 μg protein per lane (depending on the antibody), and separated by electrophoresis at 200 V until full separation as indicated by the protein ladder (PageRuler Plus Prestained Protein Ladder, #26619 or #26616, (ThermoFisher Scientific, Waltham, MA, USA). The proteins on the gel were transferred to a 0.45 μm PVDF Transfer Membrane (#88518, ThermoFisher Scientific, Waltham, MA, USA) by electroblotting (Criterion Blotter, Bio-Rad Laboratories, Hercules, CA, USA) at 20 V overnight. Blots were blocked for 1 h at room temperature using 5% nonfat dried milk powder (#A0830,0500, AppliChem, Darmstadt, Germany) and incubated overnight at 4 °C with primary antibodies: β-actin ((13E5) Rabbit mAb #4970, Cell Signaling Technology, Danvers, MA, USA), IQGAP3 (H00128239-M01 Novus Biologicals, Littleton, CO, USA), NFkB (NF-κB p65 (L8F6) Mouse mAb #6956, Cell Signaling Technology, Danvers, MA, USA), phospho-NFkB (Phospho-NF-κB p65 (Ser536) (93H1) Rabbit mAb #3033), phospho ERK1/2 (Phospho-p44/42 MAPK (Erk1/2) (Thr202/Tyr204) (D13.14.4E) XP^®^ Rabbit mAb #4370 Cell Signaling Technology, Danvers, MA, USA), phospho-p38 (Phospho-p38 MAPK (Thr180/Tyr182) (D3F9) XP^®^ Rabbit mAb #4511, Cell Signaling Technology, Danvers, MA, USA), pI3K PI3 Kinase p110α (C73F8) Rabbit mAb #4249, Cell Signaling Technology, Danvers, MA, USA), diluted in Tris-buffered saline with 5% nonfat dry milk and 0.1% Tween 20. The blots were probed with peroxidase-conjugated secondary antibodies goat-anti-rabbit or goat-anti-mouse AffiniPure antibodies (#111-035-003 or #115-035-003, Jackson ImmunoResearch Laboratories, West Grove, PA, USA)) and visualized using Clarity ECL Chemiluminescent Substrate (Bio-Rad Laboratories, Hercules, CA, USA). The blots were visualized manually using UltraCruz autoradiography film (#SC-201697, Santa Cruz Biotechnology, Santa Cruz, CA, USA) and radiography developer and fixer Renmed plus (#10850 and #10854, VIPS-MED, Fryazino, Russia), or they were visualized using a gel documentation system (ChemiScope 6000, Clinx Science Instruments Co., Ltd., Shanghai, China).

### 4.6. RNAseq Library Preparation

HaCaT_sh and HaCaT_ctrl cells were initially seeded in complete medium and 4 h later stimulated with doxycycline (Sigma-Aldrich, Merck KGaA, Darmstadt, Germany) for 24 h. Subsequently, the medium was replaced with fresh medium containing both doxycycline and pro-inflammatory psoriatic cytokines (IFNγ, TNFα, IL17) at concentrations of 20 ng/mL, 25 ng/mL, and 100 ng/mL, respectively. After 72 h of stimulation, the cells were lysed, and RNA was isolated directly from the plates using the RNEasy mini kit (Qiagen, Hilden, Germany) following the manufacturer’s instructions. The concentration of RNA was quantified using a Qubit fluorimeter with the RNA BR Assay Kit (ThermoFisher Scientific, Waltham, MA, USA). For transcriptome sequencing, libraries were prepared using the NEBNext^®^ Ultra™ II Directional kit (New England Biolabs, Ipswich, MA, USA) according to the manufacturer’s instructions. The libraries were then mixed in equimolar amounts, and their quality was assessed using a BioAnalyzer 2100 microfluidic analyzer (Agilent Santa Clara, CA, USA) with the Agilent DNA High Sensitivity Kit (Agilent Santa Clara, CA, USA).

### 4.7. RNA Sequencing and Data Analysis

The libraries were mixed and diluted to a concentration of 2 nM, and subsequently, the sequencing of the resulting library (diluted to 12 pM) was conducted on a HiSeq 2500 instrument using a 2 × 250 bp paired-end reading set with the addition of 10% Phix from PhiX Control v3 kit (Illumina, San Diego, CA, USA). The sequencing procedure utilized the HiSeq Rapid SBS Kit v2 (200) kit (Illumina, San Diego, CA, USA) according to the manufacturer’s recommendations. To assess the quality of the reads obtained through sequencing, the FastQC program was employed. Readings underwent filtering and adapter trimming using the Cutadapt program with the following parameters: —q 10.10—m 25—a AGATCGGAAGAGCACACGTCTGAACTCCAGTCAC—A AGATCGGAAGAGCGTCGTGTAGGGAAAGAGTGT—max—n 1. Adapters were removed, and reads shorter than 25 nucleotides were discarded, ensuring an average Phred score of at least 10 at each end of the read. Next, the quality reads that met the filtering criteria were mapped to the hg19 human genome using the STAR program, utilizing the annotation of the human genome version of Gencode Release 19 (GRCh37.p13). For the comparison of the expression profiles and identification of differentially expressed genes, the edgeR package was utilized. Genes with a minimum of 1 read per million in at least half of the samples were considered for each differential expression analysis. Additionally, qPCR analysis of three loci (ENSG00000183856, ENSG00000143546, ENSG00000175592) was performed to verify the differential expression. qPCR showed expression changes similar to those observed in RNAseq. Finally, pathway and gene ontology (GO) enrichment analysis were conducted using the DAVID web interface version v2023q4 [39], and transcription factor target enrichment was conducted using the ChHEA3 web interface [40].

### 4.8. Real-Time Cell Proliferation Assay

HaCaT_sh and HaCaT_ctrl cells were seeded onto xCelligence RTCA (Agilent, Santa Clara, CA, USA) 16-well plates at a density of 5000 cells per well in complete culture medium supplemented with doxycycline (1000 ng/mL). Real-time assessment of cell growth was conducted for a duration of 60 h. For the wound healing assay, cells were seeded at a density of 10,000 cells per well in complete medium containing doxycycline (1000 ng/mL). Upon reaching confluence, a sterile disposable 200 μL tip was utilized to create a scratch across the monolayer. Subsequently, debris was washed away three times with PBS, and fresh medium with the inducer was added. The cell density index was monitored dynamically over a period of 240 h (10 days), with medium replacement every 48–72 h. For the wound healing assay under pro-inflammatory conditions, cells were seeded at a density of 5000 cells per well in complete medium supplemented with doxycycline (1000 ng/mL). Upon achieving confluence, a scratch was made, followed by washing away debris with PBS. Subsequently, fresh nutrient medium containing the inducer and a mixture of pro-inflammatory cytokines (IFNγ, TNFα, and IL17 at concentrations of 20, 25, and 100 ng/mL, respectively) was added. The cell density index was evaluated dynamically over a total duration of 240 h (10 days), with replacement of the medium containing the inducer every 48–72 h.

## 5. Conclusions

Our investigation revealed that IQGAP3 knockdown inhibits several key pathways induced by cytokine stimulation, including EGFR signaling, ERK1/2 activation and death receptor-mediated apoptotic signaling, such as TNFα-TNFR signaling. Moreover, IQGAP3 knockdown affects the production of IL-6 and IFNγ and disturbs the balance between cell proliferation and apoptosis in skin cells, leading to changes in cornification and skin barrier functions. Given the crucial role of tight cell-to-cell contacts in keratinocytes, alterations in integrin-mediated signaling also contribute to the observed differences. However, the background activation of MAPK and NFkB signaling suggests that IQGAP3 is not the optimal target for therapeutic inhibition. This conclusion is also based on the diversity and number of pathways mediated by IQGAP3, indicating that targeting this protein alone is not sufficient to address the complex pathogenesis of psoriasis.

## Figures and Tables

**Table 1:** Gene ontologies enriched solely in either HaCaT_ctrl or HaCaT_sh under cytokine stimulation.

GO Enriched Only in HaCaT_Ctrl + Cyto				GO Enriched Only in HaCaT_sh + Cyto			
GO ID	Term	% of DEGs	FDR	GO ID	Term	% of DEGs	FDR
GO:0070373	negative regulation of ERK1 and ERK2 cascade	0.78	0.000178	GO:0050679	positive regulation of epithelial cell proliferation	1.45	0.010
GO:0051781	positive regulation of cell division	0.74	0.012	GO:0031099	regeneration	1.26	0.024
GO:0008625	extrinsic apoptotic signaling pathway via death domain receptors	0.67	0.006	GO:0010634	positive regulation of epithelial cell migration	1.22	0.006
GO:0006692	prostanoid metabolic process	0.47	0.013	GO:0051092	positive regulation of NF-kappa B transcription factor activity	1.15	0.005
GO:1900744	regulation of p38MAPK cascade	0.43	0.022	GO:1904019	epithelial cell apoptotic process	0.98	0.018
GO:1900745	positive regulation of p38MAPK cascade	0.34	0.020	GO:0002821	positive regulation of adaptive immune response	0.94	0.013
GO:0038134	ERBB2-EGFR signaling pathway	0.16	0.016	GO:0032649	regulation of interferon-gamma production	0.85	0.022
				GO:0007229	integrin-mediated signaling pathway	0.85	0.036
				GO:0002224	toll-like receptor signaling pathway	0.83	0.040
				GO:0032755	positive regulation of interleukin-6 production	0.72	0.038
				GO:0031424	keratinization	0.70	0.043
				GO:0032088	negative regulation of NF-kappa B transcription factor activity	0.66	0.048
				GO:0032689	negative regulation of interferon-gamma production	0.41	0.024
				GO:0010837	regulation of keratinocyte proliferation	0.41	0.038
				GO:0033561	regulation of water loss via skin	0.38	0.384
				GO:0061436	establishment of skin barrier	0.36	0.001
				GO:0043276	anoikis	0.34	0.025
				GO:0070268	cornification	0.28	0.001

## Data Availability

All authors confirm that all data and materials support their published claims and comply with field standards and are included in this article and Supplementary Materials. The datasets used and analyzed during the current study were submitted to GEO database and now are available as GSE248548.

## Supplementary Materials

The following supporting information can be downloaded at https://www.mdpi.com/article/10.3390/ijms25084545/s1.
